# Mind and body: physical health monitoring in clozapine treatment

**DOI:** 10.1192/bjo.2021.464

**Published:** 2021-06-18

**Authors:** Moataz Abdelreheem, Olivia Connell, Daniel McNally, Itunuayo Veronica Ayeni, Clare Smith

**Affiliations:** Central and North West London NHS Foundation Trust

## Abstract

**Aims:**

To evaluate physical health monitoring standards in patients on Clozapine in the community.

Standards

NICE and BNF guidelines for patients on established clozapine treatment advise annual monitoring of weight, waist circumference, pulse, blood pressure, fasting blood glucose, HbA1c, blood lipids and overall physical health assessment. Full blood count is monitored 1-4 weekly.

**Background:**

In the management of schizophrenia, antipsychotic medication remains the cornerstone of treatment. Patients affected carry a significant physical health burden with a reduced life expectancy of 10-25 years. Factors that contribute include sedentary lifestyles, consequent obesity and cardiovascular disease, disengagement from health services, a higher incidence of suicide and the physical side effects of antipsychotic medication. For these reasons, comprehensive routine physical assessment of patients on antipsychotic treatment is of central importance.

**Method:**

This audit is a retrospective study of patients known to South Kensington & Chelsea Community Mental Health Team (CMHT). Patients (n = 48) were audited from the Clozapine clinic SystemOne database over a one year period (October 2018-2019) to assess annual monitoring of full blood count (FBC), urea and electrolytes (U&Es), lipid profile, liver function tests (LFTs), HbA1C, thyroid function tests (TFTs), clozapine levels, ECG, and general physical and mental health review.

**Result:**

Of the 48 patients, one was transferred to a different service so was excluded (n = 47 total).

All (100.0%) of the patients had annual FBC tests. All but one (97.9%) of the patients had a physical health review including blood pressure, pulse, weight and BMI measurement. Three quarters (74.5%) received annual U&Es and LFTs. Almost two thirds of patients had annual lipid and HBA1c screening (63.8%) and over half the cohort had annual TFTs (61.7%). Regarding annual multidisciplinary mental health review, this was performed for the majority of the patients (70.2%).

Contrastingly, only a quarter of the patients received annual screening of glucose and Clozapine levels (27.7% for both). Only 12 patients had annual ECG (25.5%).

**Conclusion:**

Following review it is clear that most parameters were monitored annually in a majority of patients. However, shortcomings were detected, specifically annual ECG and waist circumference monitoring.

In order to ensure comprehensive monitoring of mental and physical health of patients on Clozapine, flow charts of tests and reviews needed for each patient were written up clearly and will be included in the management pathway for every patient on Clozapine. This was agreed to minimise missing any step, particularly annual ECGs.

